# Language assessment in primary progressive aphasia: Which components should be tested?

**DOI:** 10.1371/journal.pone.0318155

**Published:** 2025-02-05

**Authors:** Andressa Aguiar da Silva, Marcela Lima Silagi, Karin Zazo Ortiz

**Affiliations:** Department of Language, Speech and Hearing Sciences, Universidade Federal de São Paulo (UNIFESP), São Paulo, SP, Brazil; IRCCS San Raffaele Scientific Research Institute, ITALY

## Abstract

**Introduction:**

Primary progressive aphasia (PPA) is a dementia syndrome whose onset and course manifests with language deficits. There is a lack of instruments for clinical assessment of language in dementia and further research in the area is needed. Therefore, the objective of the present study was to identify language tasks that can aid the process of clinically diagnosing PPA and to determine those tasks most impaired in this population.

**Method:**

A sample of 87 individuals comprising 2 groups was assessed: a PPA group (PPAG) of 29 PPA patients; and a control group (CG) of 58 healthy subjects matched for age and education. All participants underwent a brief cognitive battery followed by a comprehensive language assessment using the MTL-BR Battery.

**Results:**

A statistically significant performance difference was found between the PPAG and CG on the following tasks: structured interview, oral comprehension of phrases, oral narrative discourse, written comprehension of phrases, written dictation, sentence repetition, semantic verbal fluency, oral naming of nouns and verbs, object manipulation, phonological verbal fluency, body part recognition and left-right orientation, written naming of nouns, oral text comprehension, number dictation, written narrative discourse, written text comprehension and numerical calculations (mental and written).

**Conclusion:**

The results revealed that performance of PPA patients was poorer compared to healthy subjects on various language tasks. The most useful subtests from the MTL-BR battery for aiding clinical diagnosis of PPA were identified, tasks which should be prioritized when assessing this patient group.

## Introduction

Primary progressive aphasia (PPA) is a dementia syndrome marked by a gradual loss in communication functioning due to the effects of degeneration on brain networks involved in speech and language processing [1]. This dementia syndrome, can have different causes such as frontotemporal lobar degeneration (FTLD) or Alzheimer´s Disease (AD) has several classical variants, each of which has specific language features.

The different variants of PPA present some typical and predominant linguistic characteristics but there is a wide range of symptom possibilities in addition to those mentioned. Identifying these variants early can improve patients’ quality of life. For instance, the semantic PPA (sv PPA) can be characterized by anomia, evident in discourse and on visual confrontation naming tasks, and by difficulty understanding individual words, deficits associated with gradual loss of semantic knowledge. The non-fluent/agrammatic variant (nfav PPA) is characterized by agrammatism, effortful hesitant speech, as well as phonemic and phonological deficits consistent with apraxia of speech. The logopenic variant (lv PPA) is marked by naming difficulties due to lexical access failures, with the occurrence of anomia in discourse and failures in phrase repetition stemming from deficits in the phonological loop of working memory [1–3]. In addition to these variants, there is also the mixed/unclassifiable form of the disease. These patients do not fit the profiles of any of variants because they either do not have any of the core features of any specific variant or they have more symptoms of the classical variants and in this case the individuals may manifest classic characteristics of more than one subtype [4] precluding a clear diagnosis [5,6].

The language disorder might depend either on the intrinsic deterioration of the linguistic system or on a more general cognitive decline and seem to be the result of an interplay between both. In this sense, the mean of language assessment in overall dementias becomes crucial.

PPA is a rare disease that is complex to diagnose due to the heterogeneous presentation of cases. Despite the importance of language with regard to making differential diagnoses of neurodegenerative diseases, there is still a lack of language assessment tools for all types of dementia, including PPA. Instruments originally developed for assessing post-stroke aphasia are still widely used for diagnosing dementias [7]. Although tests specifically developed for assessing aphasia are not best suited for evaluating language deficits associated with neurodegenerative diseases [8,9], some aphasia symptoms secondary to acute lesions are similar to those seen in PPA [10]. More recently, a number of tests have been proposed [11,12], and some retrospective studies, with large PPA patients have already demonstrated that some combined language tasks are useful to identify PPA and distinguishing patients’ subtype syndrome [13], even though in very early stages from the disease [4] that can help to guide linguistic-cognitive interventions.

In this sense it is also worth bearing in mind that tests should undergo full psychometric analysis, including tests of validity, internal consistency, and reliability [14]. The Montreal Toulouse Language-Brasil Battery (MTL-BR) [15] is the only instrument for language assessment available which normative data in Brazil. It was mainly designed to assess aphasia, then it is important to understand if its tasks (including its stimulus) are useful and suitable for assessing language disorders due to degenerative cases. Moreover, in this context, brief batteries are also needed, since the use of extended batteries are often impractical to implement [13] and ascertaining which language assessment tasks can best identify PPA can be valuable

In view of how some instruments or language tasks can best discriminate PPA the present study sought to identify, based on a comprehensive language assessment - MTL-BR -which language tasks can aid identifying the most and/or early impaired language processing in this population.

## Materials and methods

The present study was conducted at the Department of Language, Speech and Hearing Sciences of the Universidade Federal de São Paulo (UNIFESP) – Escola Paulista de Medicina. The study was approved by the local Research Ethics Committee under permit no. 0540/2022. Participants were enrolled onto the study on a voluntary basis after signing the Free and Informed Consent Form, devised in compliance with the recommendations of the National Board of Health (CNS) pursuant to Resolution Ruling 466/12 of the CNS/MS (Ministry of Health).

### Participants

The study sample comprised two groups: an aphasia group of patients neurologically diagnosed with primary progressive aphasia (PPAG) and a control group of healthy subjects (CG) matched with patients for age and education.

For the PPAG, inclusion criteria were patients referred to neurologists specialized in dementia syndromes, with suspicion of having PPA, guided by the previously outlined criteria [1]. All patients were exposed to a neurological and a neuropsychological evaluation and had undergone a neuroimaging scan that differentially diagnose variant via location and severity of atrophy. The data from all examinations also allowed the differential diagnosis between PPA and other dementias. Individuals at the severe stage of dementia, those with less than 12 points on the Mini-Mental State Examination (MMSE) [16] or those with the absence of speech or inability to complete the evaluation were not included, based on the premise that participants with this status would be unable to complete the required procedures.

A convenience sample of healthy older volunteers without neurological disorders or a history of psychiatric issues was included. The specific inclusion criteria for the CG included normal performance on cognitive screening tests.

The exclusion criteria for the study participants were previous or current use of alcohol or illicit drugs; use of psychoactive drugs other than atypical neuroleptics and antidepressants in the past month; previous history of severe neurological or psychiatric disorders (i.e., epilepsy, cancer, and schizophrenia); visual changes and hearing loss, absence of verbal expression and complaint of predominant speech or language difficulties. This information was collected by applying a questionnaire.

The PPAG comprised 29 patients while the CG contained 58 healthy subjects, all of whom met the criteria for inclusion in the study.

The participants were patients assessed at the outpatient clinic of the Language and Speech Pathology division of Sao Paulo Federal University and mainly their relatives and caregivers formed the CG.

The participants from this study were assessed between February, 1 st, 2023 up to the last of July.

### Procedures

All participants that met the inclusion criteria underwent a neuropsychological evaluation. Cognitive screening tests and a neuropsychological battery were applied. The MMSE was used as a screening tool. The Portuguese translation and scoring of the MMSE was adopted [17] with cut-off scores adapted to the subjects’ educational levels: 9 to 11 years of education = 28; and highly educated (more than 12 years) = 29.The brief cognitive battery comprised the Trail-Making Test [18], the forward and backwards Digit Span test [19], Corsi Block Test [20–22], verbal memory test from the CERAD Battery [23], and the Clock-Drawing Test [24].

The tests were applied just to determine the overall cognitive status of the patients.

Then, all participants were administered the Montreal-Toulouse Language Assessment Battery (MTL-BR Battery) [15]. This test was chosen because it provides a broad assessment of auditory/oral and written comprehension and oral and written production and is the only validated test for acquired language disorders in Brazilian Portuguese. The test was applied according to the instructions contained in the application manual and comprises 22 subtests as outlined below:

1. Structured interview: includes 13 open-ended questions to analyze speech and auditory comprehension. 13 items with maximum score of two points each. Total score ranges from 0 to 26 points.
2. Automatic speech: assesses the ability to evoke automatisms, such as numbers, days of the week and the birthday song, evaluated for form and content. Total score ranges from 0 to 6 points.
3. Oral comprehension: measures the ability to identify images that represent words and phrases from auditory input. The task consists of a total of 19 items, five words (boards with six stimuli comprising one target and five distracters: one phonological, one semantic, one visual and two neutral) and 14 sentences.The maximum score is five points for words and 14 points for phrases, with one point for each correct answer. Total score ranges from 0 to 19 points.
4. Oral narrative discourse: evaluates the ability to tell a story from visual inputs. The task consists of describing a picture depicting a bank robbery. The narrative is analyzed for the number of words and the number of information units (IUs) produced. They IUs were considerate the informative and relevant elements that are present in an organized discourse structure and the IUs expected were: bank, robbery, thieves, guard, car, running, waiting, calling, people and money. Each IU gets point. The maximum score for IUs is 10 points.
5. Written comprehension: assesses the written comprehension of words and phrases. The task consists of a total of 13 items, five words (boards with six stimuli comprising one target and five distracters: one graphemic, one semantic, one visual and two neutral) and 8 sentences. One point is given for each correct answer, with total task score ranging from 0 to 13 points.
6. Copying: a sentence must be copied while changing the allographic form. Maximum task score is 8 points.
7. Written dictation: assesses the participant’s ability to write dictated words and phrases. The task consists of 9 items (5 regular and 3 irregular words and 1 non-word) and three sentences. The maximum scores are 9 and 13 points for words and phrases respectively, with one point is given for each word written correctly, yielding a maximum score of a 22 points.
8. Repetition: measures the ability to reproduce the auditory stimuli provided. The task consists of 11 items (8 words and 3 non-words) and three sentences. The maximum scores are 11 and 22 points for words and phrases respectively, with one point for each word repeated correctly, yielding a maximum score of a 33 points.
9. Reading aloud: assesses reading of words and phrases. The task consists of 12 items (3 irregular and 5 regular words and 3 non-words) and three sentences. The maximum scores are 11 and 22 points for words and phrases respectively, with one point for each word repeated correctly, yielding a maximum score of a 33 points.
10. Semantic verbal fluency: evaluates the ability for lexical production of words from the animal’s semantic category within 90 seconds. One point is given for each correctly produced word.
11. Non-verbal praxis: assesses the ability to produce isolated gestures and movement sequences involving the face and tongue, requested by the evaluator through verbal instructions. The task consists of a total of 6 items with maximum scores of 4 points each, giving a maximum total of 24 points.
12. Naming: measures lexical access using pictures that refer to nouns and verbs. Fifteen pictures are presented (12 nouns and three actions), placed on individual boards. The maximum score is 30 points, comprising 15 items with a maximum score of two points each. The criteria for scoring is incorrect answer: zero; item semantic related or description: 1 point; and correct answer: 2 points. Score on this task ranges from 0 to 30 points.
13. Object manipulation by verbal command: assesses the ability to understand simple and complex verbal commands. The individual is instructed to perform six commands given by the clinician, using physical objects (key, comb, cup, pen, and paper). The complexity of orders increases gradually. One point is given to each part of the command that is properly performed by the individual. This task is scored from 0 to 16 points.
14. Phonological verbal fluency: evaluates the number of words produced beginning with the letter M within 90 seconds. A point is given for each word produced.
15. Body part recognition and left-right orientation: assesses recognition of parts of the body and laterality orientation. The maximum score is eight points, of which four points are given for each body part (limbs) and the other four are given for the right-left orientation.
16. Written naming: fifteen pictures are presented (12 nouns and three actions), placed on individual boards. The maximum score is 30 points, comprising 15 items with a maximum score of two points each. The criteria for scoring is incorrect answer: zero; item semantic related or description: 1 point; and correct answer: 2 points. Score ranges from 0 to 30 points.
17. Oral text comprehension: assesses the ability to understand auditory input from a text read by the clinician. The individual must answer six questions orally after listening to the text (three open-ended and three closed-ended questions). A maximum of two points for each of the three open-ended questions and one point for each of the closed-ended questions Scores on this subtest range from 0 to 9 points.
18. Number dictation: assesses the ability to trans- code six numbers from auditory stimuli to written form. Score ranges from 0 to 6 points.
19. Reading of numbers: assesses the ability to recognize six Arabic numerals and reproduce them orally. Score ranges from 0 to 6 points.
20. Written narrative discourse: involves the ability to write a story from visual input. The task consists of a picture depicting a robbery at a bakery. We analyzed the number of words and the number of information units (IUs) produced. They IUs were considerate the informative and relevant elements that are present in an organized discourse structure and the IUs expected for this picture were: bakery, robbery, robbers, guard, car, running, waiting, calling and gun (one point for each word). The maximum score is 10 points for IUs.
21. Written text comprehension: assesses the ability to understand a written text. The individual must answer six questions orally after reading the text (three open-ended and three closed-ended questions). A maximum of two points for each of the three open-ended questions and one point for each of the closed-ended questions Score on this task ranges from 0 to 9 points.
22. Numerical calculation: evaluates the ability to perform mathematical operations of addition, subtraction, multiplication and division, as well as mental simple mathematical problem. Each subtest – mental and written calculations – is scored from 0 to 6 points.

### Statistical analysis

For the descriptive analysis, all demographic variables and performance on the MTL-BR subtests were expressed as mean, standard deviations and range.

Given the Gaussian distribution of the data, comparison between means was carried out using parametric and non-parametric tests.

Statistical treatment was performed using Student´s *t-*test for independent samples. Welch correction was used to calculate *p*-value, where applicable.

The level of statistical significance adopted was 5%. The software SPSS Statistics, version 28.0 (IBM Corp., Armonk, NY, USA) was used for all statistical analyses.

## Results

### Sample characteristics

The characteristics of the study sample regarding age and years of education are presented in Table 1.

**Table 1:** Descriptive values and comparative analysis of PPA and Control groups according to age and education.

	Group	n	Mean	SD	Median	Min.	Max.	p	ST.
Age (years)	PPA	29	68.41	7.80	69.00	56.00	87.00	0.808	0.055
	Control	58	67.97	8.31	65.50	55.00	88.00		
Education level (years)	PPA	29	15.97	2.04	16.00	11.00	22.00	0.016*	0.491
	Control	58	14.66	2.93	15.00	9.00	23.00		

The results given in Table 1 show a statistically significant difference between the CG and PPAG for education. Although the controls were matched by convenience, with the selection of subjects who had a similar education to patients, a small difference between PPA and Control groups was found (15.97 *versus* 14.66 years of formal education), but it must be highlighted that it does not seem that this difference could interfere on the results obtained, first because the group with more years of education was the PPA and mainly because there is no difference in performing MTL-BR tasks in subjects with more than 8 years of formal education [14].

There was no statistically significant group difference for age.

Of the 29 patients in the PPAG, 13 exhibited the semantic variant, 11 the logopenic variant, 4 non-fluent and/or agrammatic variant, and 1 had the mixed variant. Regarding performance on the MMSE, the PPA group had a mean score of 23.33 (SD ± 3.70), while the CG had a mean score of 29.17 (SD ± 3.43). Considering the MMSE scores 12 PPA patients were at moderate stage and 17 at mild stage of the disease. Mean time since PPA diagnosis was 30 (SD ± 14.02) months.

The results for the comparison of performance by the PPAG and CG on the MTL-BR Battery subtests are presented in Table 2.

**Table 2:** Descriptive values and comparative analysis of PPA and Control groups according to performance on subtests of MTL-BR Battery.

MTL-BR	Group	Mean	SD	Median	Min.	Max.	P
Structured interview	PPA	23.31	3.64	25.00	13.00	26.00	0.015*
	Control	25.90	0.36	26.00	24.00	26.00	
Oral comprehension – words	PPA	4.64	0.83	5.00	2.00	5.00	0.104
	Control	4.97	0.18	5.00	4.00	5.00	
Oral comprehension – phrases	PPA	11.43	2.18	12.00	7.00	14.00	0.002*
	Control	13.19	1.10	14.00	10.00	14.00	
Oral narrative discourse - total numbers of words	PPA	66.48	44.38	52.00	19.00	161.00	0.16
	Control	54.09	20.38	52.00	14.00	105.00	
Oral narrative discourse –information unit	PPA	5.03	2.18	5.00	1.00	9.00	< 0.001*
	Control	6.91	1.81	7.00	2.00	10.00	
Oral narrative discourse - elements	PPA	2.14	0.79	2.00	0.00	3.00	0.034*
	Control	2.50	0.68	3.00	1.00	3.00	
Written comprehension - words	PPA	4.75	0.65	5.00	2.00	5.00	0.120
	Control	5.00	0.00	5.00	5.00	5.00	
Written comprehension - phrases	PPA	6.61	1.73	7.00	2.00	8.00	0.011*
	Control	7.65	0.64	8.00	6.00	8.00	
Copying	PPA	7.74	0.81	8.00	5.00	8.00	0.594
	Control	7.84	0.62	8.00	4.00	8.00	
Written dictation	PPA	18.63	3.36	20.00	11.00	22.00	0.005*
	Control	21.12	1.82	22.00	11.00	23.00	
Repetition – words	PPA	10.50	1.04	11.00	7.00	11.00	0.202
	Control	10.78	0.53	11.00	9.00	11.00	
Repetition- phrases	PPA	18.32	5.06	21.50	7.00	22.00	0.006*
	Control	21.95	0.22	22.00	21.00	22.00	
Reading aloud – words	PPA	11.24	1.27	12.00	6.00	12.00	0.198
	Control	11.59	0.88	12.00	7.00	12.00	
Reading aloud – phrases	PPA	20.72	0.59	21.00	19.00	21.00	0.064
	Control	20.95	0.29	21.00	19.00	21.00	
Semantic verbal fluency	PPA	10.62	6.24	10.00	1.00	21.00	< 0.001*
	Control	23.91	5.80	23.00	12.00	39.00	
Non-verbal praxis	PPA	22.70	2.95	24.00	12.00	24.00	0.159
	Control	23.86	0.48	24.00	22.00	24.00	
Naming – nouns	PPA	19.34	4.83	20.00	6.00	24.00	0.003*
	Control	23.69	0.65	24.00	21.00	24.00	
Naming – verbs	PPA	5.69	0.60	6.00	4.00	6.00	0.038*
	Control	5.97	0.26	6.00	4.00	6.00	
Object manipulation by verbal command	PPA	14.10	2.73	16.00	8.00	16.00	0.024*
	Control	15.83	0.50	16.00	14.00	16.00	
Phonological verbal fluency	PPA	8.21	5.42	7.00	0.00	20.00	< 0.001*
	Control	17.69	5.77	17.00	5.00	31.00	
Body part recognition and left-right orientation	PPA	8.00	0.00	8.00	8.00	8.00	0.034*
	Control	7.97	0.18	8.00	7.00	8.00	
Written naming – nouns	PPA	19.04	6.41	22.00	2.00	24.00	0.029*
	Control	22.33	1.44	22.00	18.00	24.00	
Written naming – verbs	PPA	5.82	0.48	6.00	4.00	6.00	0.218
	Control	5.95	0.29	6.00	4.00	6.00	
Oral text comprehension	PPA	4.45	2.26	4.00	1.00	9.00	< 0.001*
	Control	7.97	1.23	8.00	4.00	9.00	
Number dictation	PPA	5.00	1.65	6.00	0.00	6.00	0.025*
	Control	5.95	0.29	6.00	4.00	6.00	
Reading of numbers	PPA	5.70	0.72	6.00	3.00	6.00	0.105
	Control	6.00	0.00	6.00	6.00	6.00	
Written narrative discourse – total numbers of words	PPA	34.24	19.58	32.00	0.00	76.00	0.427
	Control	37.76	18.31	39.00	7.00	95.00	
Written narrative discourse - information unit	PPA	6.07	2.55	6.00	0.00	10.00	0.186
	Control	5.24	3.00	6.00	1.00	10.00	
Written Narrative Discourse- elements	PPA	2.21	0.83	2.00	0.00	3.00	0.005*
	Control	3.36	2.49	3.00	1.00	9.00	
Written Text Comprehension	PPA	5.86	2.85	6.00	0.00	9.00	< 0.001*
	Control	8.57	0.73	9.00	6.00	9.00	
Numerical Calculation – mental	PPA	4.31	2.09	5.00	0.00	6.00	0.010*
	Control	5.43	0.88	6.00	3.00	6.00	
Numerical Calculation – written	PPA	4.48	2.02	5.00	0.00	6.00	0.016*
	Control	5.74	0.85	6.00	1.00	6.00	

ST, Student´s *t*-test for independent samples; SD, Standard deviation; Min, Minimum; Max, Maximum; *, statistically significant value (*p* ≤  0.05).

The results revealed a statistically significant difference in performance between the PPAG and CG on the following tasks of the MTL-BR Battery: structured interview, oral comprehension of phrases, oral narrative discourse, written comprehension of phrases, written dictation, sentence repetition, semantic verbal fluency, oral naming of nouns and verbs, object manipulation, phonological verbal fluency, body part recognition and left-right orientation, written naming of nouns, oral text comprehension, number dictation, written narrative discourse, written text comprehension and numerical calculations (mental and written). No statistically significant differences between the PPAG and CG were evident for the other tasks of the MTL-BR Battery.

## Discussion

The results of the present study, as expected, showed that performance of the PPAG was worse than the CG on all the language tasks assessed by the MTL-Brasil Battery.

The tasks from MTL- BR Battery allow that many linguistic processing can be investigated and compared. It is very important because language processing is the expression of a complex cognition function and each linguistic task can interact with working and long-term memory, attentional skills, and executive control in different ways [25]. In this sense, understanding the deterioration of the linguistic system can help to establish compensatory mechanisms in an attempt to preserve the communication.

There was a statistically significant difference in performance on the structured interview, oral comprehension of phrases and text, oral narrative discourse, object manipulation, body part recognition and left-right orientation, sentence repetition, oral naming of nouns and verbs, phonological and semantic verbal fluency, written comprehension of phrases and text, written naming of nouns, written dictation, number dictation, and numerical calculations mentally and on paper (Table 2). Our results suggest that maybe these tasks could be considerate for language screening tasks in this population. These findings will be further discussed below. It was also found that the PPAG produced a discourse containing a greater number of words compared to the CG. A higher number of words is not necessarily linked to a better quality of an oral narrative since a higher wordiness or verbosity [26] can be associated with linguistic decline.

Regarding to oral comprehension, the impaired performance on phrases can be expected in PPA because, particularly for the agrammatic/non-fluent variant, patients have difficulty performing syntactic processing [27,28], especially for the non-canonic structures incorporated in the sentences from the MTL-Brasil Battery. In the logopenic variant, this same difficulty may be associated with deterioration of phonological working memory, which influences comprehension of long sentences made up of different syntactic structures [28–30] and although the typical difficulty with svPPA is understanding words, these patients commonly have impaired sentence comprehension as the disease progresses. Similarly, on the object manipulation task by verbal command, the higher the number of command items, the greater the overload of working memory. Likewise, this effect also occurs on the body-part recognition and left-right orientation task, whereby the individual has to process two items of information at the same time (body part plus side). However, when identifying body parts and notions of left and right, difficulties involving semantic recognition may also occur. In fact, a previous study found impaired body-part and body-side identification in AD patients, where the spatial orientation involved in this comprehension task can be difficult for some patients with dementia [9].

Oral comprehension difficulties were also found on the structured interview task. The structured interview task from the MTL-Brasil Battery consists of both simple and complex questions, distributed hierarchically. The simpler questions require comprehension of elements of everyday life and are therefore easier to understand. However, the more complex questions involve analysis of situations about which the examinee is asked their opinion and, therefore, demands more cooperation between language and other cognitive functions, particularly executive function. Similarly, comprehension of text is a complex task that requires interaction of several cognitive domains besides language, especially episodic memory, working memory and attention, together with the demands of oral language and executive functions for retelling the story [27,31]. It is worth mentioning that no statistically significant difference was found between groups considering comprehension of words. Although thirteen patients exhibited the semantic variant, most of them were at mild stage and it seems that MTL stimuli from this task was very familiar and easy for them.

Regarding oral production tasks, difficulties were observed on phrase repetition, possibly due to phonological or grammatical deficits, or associated with apraxia speech. Phrase repetition is one of the main tasks used for identifying logopenic PPA due to failure in short-term phonological memory [32]. In non-fluent/agrammatic PPA, this deficit stems mainly from impaired motor planning of speech which leads to apraxia of speech [33]. However, repetition problems can also be caused by a deficit in syntactic processing [34].

With regard to oral naming of words, this task is especially important in assessing both the semantic [35] and logopenic variants of PPA, due to their associated lexical-semantic or lexical-phonologic deficits [36,37]. In semantic PPA, anomia manifests due to gradual deterioration of semantic knowledge. For logopenic PPA, there are more failures in lexical access, while semantic knowledge tends to remain preserved until moderate stages of the disease [38]. In non-fluent/agrammatic PPA, naming of verbs can be more impacted than nouns [39,40]. Although the stimuli from MTL-BR battery are considerate very easy, in the present study they were able to discriminate groups.

Regarding oral narrative discourse, analysis of language in connected speech provides different information, which is particularly relevant in dementia due to the broad cognitive nature of the disorder. To produce discourse, a speaker must integrate linguistic and non-linguistic skills. For this reason, discourse analysis is seen as a sensitive tool for detecting language impairment [41]. In the MTL-BR battery, discourse production takes place based on the input of a figure. Thus, the task requires adequate visual perception and visual inference, where these elements provide the basis for producing a narrative. The results in Table 2 show that the PPAG produced a discourse containing a greater number of words compared to the CG, but with fewer information units. This finding can be explained by the presence of circumlocutions which occur primarily in semantic PPA and secondarily in logopenic PPA. In other words, despite the presence of anomia, semantic and verbal paraphasias, besides substitution of words with unspecific generic terms, participants with PPA seek to maintain a narrative discourse by repeating ideas and talking around the topic [2,3,42,43]. Moreover, deficits such as agrammatism, morphemic and phonemic paraphasias commonly found in nfav PPA [27] also affect the ability to produce discourse.

Impaired performance on the semantic verbal fluency and phonological tasks were expected given that lexical access issues are common to all dementia syndromes [2,44,45]. Semantic verbal fluency measures the integrity of semantic memory, whereas phonologic verbal fluency is a more sensitive measure of executive dysfunctions and impaired phonologic processing [46]. In this respect, the present study findings corroborate the results of previous studies showing the important role of these tasks in language assessment. Thus, evidence suggests that semantic verbal fluency may be more impaired than phonologic fluency in individuals with svPPA. Conversely, phonological fluency may be more impaired than semantic verbal fluency in patients with nfavPPA. In lvPPA, the evidence is conflicting, with some studies reporting similar performance on both tasks [46,47], while others show greater impairment in phonologic fluency [48].

Considering written comprehension tasks, differences were found between groups on comprehension of phrases and of written text. In the case of comprehension of phrases, dissociation between the lexical and morphosyntactic domains may hamper decoding of grammatical structure [49]. For written comprehension of text, besides the language difficulties outlined, performing this task involves greater cognitive demand, such as information retention, short-term memory, working memory and attention. Moreover, although language deficits predominate in PPAs, patients already exhibited impairments to several cognitive domains, with consequent influence on performing highly demanding tasks.

Regarding written production tasks, compared to CG subjects, patients from the PPAG had lower performance for word dictation, written naming and narrative discourse production.

Writing difficulties are common in individuals with PPA [1], such as central dysgraphia, which can impair word spelling, while difficulties performing grapheme-phoneme conversion and peripheral dysgraphia, such as grapheme buffer failure [50] can occur, with a greater incidence of peripheral errors than central errors in some cases [51].

The word dictation task of the MTL-BR battery consists of non-words together with both regular and irregular words. Hence, this task involves a number of writing subsystems of the lexical and phonological routes, which may be impaired in different variants of PPA [50,52–54]. In addition, the association of central dysgraphia with peripheral components is not uncommon in svPPA [50,55].

On the written naming task, specific deficits caused by the lexical and phonological routes are associated with failures in lexical-semantic access.

In written narrative discourse, unlike in oral narrative discourse, there was a statistically significant difference between the two groups only for the elements making up the scene (robbery at the bakery). Written text production may require more planning time, and there is more opportunity for formulating and reformulating ideas [56], factors which likely contributed to better performance on this task relative to the oral discourse production. Moreover, difficulties beyond the specific writing disorders already mentioned were expected because it is known that producing discourse is a complex task that involves retrieving information from memory and deciding on which elements should be included or excluded, entailing major interaction among cognitive functions.

No group difference was detected on the non-verbal praxis tasks. Although the literature shows that deficits on this test can be expected in patients with agrammatic PPA [33,57], the presence of motor difficulties is relative [58]. In the present sample, only 4 patients had the agrammatic variant, which can develop with apraxia. Furthermore, the non-verbal praxis task, in the manner executed in the battery, without repetitive gestures or time control, appears to have low sensitivity for detecting more subtle motor impairments.

Regarding to numerical knowledge and calculation, the PPAG had poorer performance than the CG on tasks such as number dictation and mental calculations. In fact, the transcoding tasks, since they involve language processing, may be especially hard for people with language deficits [59], while dyscalculia may also be present, seen in both acute lesions and PPA [59,60].

Lastly, although the MTL-BR battery seems to be an excellent instrument for language assessment, as all instruments, it has limitations. As a battery that was originally design for persons with aphasia some stimuli from some tasks, as pointed out before, are easy. At this point, some tasks cannot be so good to differentiate deficits in a very early beginning of the disease and/or for highly educated individuals.

### Study limitations

Although the tasks that differentiate PPA patients from healthy subjects were identified using the MTL-BR, further studies involving larger samples should be conducted to investigate whether these tasks can distinguish between PPA variants.

In addition, future studies are warranted exploring potential correlations of language deficits and decline in other cognitive functions and dementia staging.

## Conclusions

This study identified some differences in language processing in PPA patients. Language changes should be monitored and could make us better understand how compensatory strategies aiding communication could be implemented. In this sense, an overview of language impairment can be helpful.

Therefore, the tasks that PPA patients had poorer performance at MTL-BR such as structured interview, oral comprehension of phrases, oral narrative discourse, written comprehension of phrases, written dictation, repetition of phrases, semantic verbal fluency, naming nouns and verbs, object manipulation by verbal command, phonological verbal fluency, body part recognition and left-right orientation, written naming of nouns, oral text comprehension, number dictation, written text comprehension and numerical calculation (mental and written) should be prioritized in assessments of patients with PPA and incorporated into brief tools for use in routine neurologic exams and for diagnosis of PPA.

## Supporting information

S1 Table CG and PPAG data. MD = Missing Data.
(XLSX)

## References

[1] Gorno-Tempini ML, Hillis AE, Weintraub S, Kertesz A, Mendez MF, Cappa SF. Classification of primary progressive aphasia and its variants. Neurology. 2011;76(11):1006–14. 21325651 10.1212/WNL.0b013e31821103e6 PMC3059138

[2] Mesulam M-M, Rogalski EJ, Wieneke C, Hurley RS, Geula C, Bigio EH, et al. Primary progressive aphasia and the evolving neurology of the language network. Nat Rev Neurol. 2014;10(10):554–69. doi: 10.1038/nrneurol.2014.159 25179257 PMC4201050

[3] Marshall CR, Hardy CJD, Volkmer A, Russell LL, Bond RL, Fletcher PD, et al. Primary progressive aphasia: a clinical approach. J Neurol. 2018;265(6):1474–90. doi: 10.1007/s00415-018-8762-6 29392464 PMC5990560

[4] Stockbridge MD, Tippett DC, Breining BL, Hillis AE. When words first fail: Predicting the emergence of primary progressive aphasia variants from unclassifiable anomic performance in early disease. Aphasiology. 2023;37(8):1173–85. doi: 10.1080/02687038.2022.2084706 37377938 PMC10292722

[5] Senaha MLH, Caramelli P, Brucki SMD, Smid J, Takada LT, Porto CS, et al. Primary progressive aphasia: classification of variants in 100 consecutive Brazilian cases. Dement Neuropsychol. 2013;7(1):110–21. doi: 10.1590/S1980-57642013DN70100017 29213827 PMC5619553

[6] Utianski RL, Botha H, Martin PR, Schwarz CG, Duffy JR, Clark HM, et al. Clinical and neuroimaging characteristics of clinically unclassifiable primary progressive aphasia. Brain Lang. 2019;197:104676. doi: 10.1016/j.bandl.2019.104676 31419589 PMC6726500

[7] Battista P, Miozzo A, Piccininni M, Catricalà E, Capozzo R, Tortelli R. Primary progressive aphasia: a review of neuropsychological tests for the assessment of 23 speech and language disorders.. Aphasiology. 2017;31(12):1359–78.

[8] Clark H, Utianski R, Duffy J, Strand E, Botha H, Josephs K. Western aphasia battery–revised profiles in primary progressive aphasia and primary progressive apraxia of speech. Am J Speech Lang Pathol. 2020;29(1S):498–510. 31639312 10.1044/2019_AJSLP-CAC48-18-0217 PMC7233113

[9] Ortiz KZ, DE Lira JO, Minett TSC, Bertolucci PHF. Language impairment in the moderate stage of dementia due to Alzheimer’s disease. Arq Neuropsiquiatr. 2021;79(4):283–9. doi: 10.1590/0004-282X-ANP-2020-0123 34133508 PMC9231441

[10] Grossman M, Irwin DJ. Primary Progressive Aphasia and Stroke Aphasia. Continuum (Minneap Minn). 2018;24(3):745–67. doi: 10.1212/CON.0000000000000618 29851876 PMC7988735

[11] Chien Y-W, Hong S-Y, Cheah W-T, Yao L-H, Chang Y-L, Fu L-C. An automatic assessment system for Alzheimer’s disease based on speech using feature sequence generator and recurrent neural network. Sci Rep. 2019;9(1):19597. doi: 10.1038/s41598-019-56020-x 31862920 PMC6925285

[12] Macoir J, Fossard M, Lefebvre L, Monetta L, Renard A, Tran TM, et al. Detection test for language impairments in adults and the aged-a new screening test for language impairment associated with neurodegenerative diseases: validation and normative data. Am J Alzheimers Dis Other Demen. 2017;32(7):382–92. doi: 10.1177/1533317517715905 28639484 PMC10852687

[13] Stockbridge MD, Tippett DC, Breining BL, Vitti E, Hillis AE. Task performance to discriminate among variants of primary progressive aphasia. Cortex. 2021;145:201–11. doi: 10.1016/j.cortex.2021.09.015 34742101 PMC8633174

[14] Pagliarin KC, Ortiz KZ, Parente MA de MP, Nespoulous J-L, Joanette Y, Fonseca RP. Individual and sociocultural influences on language processing as assessed by the MTL-BR Battery. Aphasiology. 2014;28(10):1244–57. doi: 10.1080/02687038.2014.918573

[15] Parente M, Ortiz KZ, Soares ECS, Scherer L, Fonseca R, Joanette Y, et al. Bateria Montreal-Toulouse de Avaliação da Linguagem-Bateria MTL-Brasil. São Paulo: Vetor Editora; 2016.

[16] Folstein MF, Folstein SE, McHugh PR. “Mini-mental state”. a practical method for grading the cognitive state of patients for the clinician. J Psychiatr Res. 1975;12(3):189–98. doi: 10.1016/0022-3956(75)90026-6 1202204

[17] Brucki SMD, Nitrini R, Caramelli P, Bertolucci PHF, Okamoto IH. Suggestions for utilization of the mini-mental state examination in Brazil. Arq Neuropsiquiatr. 2003;61(3B):777–81. doi: 10.1590/s0004-282x2003000500014 14595482

[18] Strauss E, Spreen O, Sherman EMS. A compendium of neuropsychological tests: Administration, norms and commentary. 2 ed. New York: Oxford University Press; 1998.

[19] Antunes AM, Costa AJ, Haase VG. Compêndio de testes neuropsicológicos: atenção, funções executivas e memória. Em: Costa AJ, Moura RJ, Haase VG, organizadores. Compêndio de Testes Neuropsicológicos: Atenção, Funções Executivas e Memória. Editora Hogrefe; 2017.

[20] Campanholo KR, Romão MA, Machado M de AR, Serrao VT, Coutinho DGC, Benute GRG, et al. Performance of an adult Brazilian sample on the trail making test and stroop test. Dement Neuropsychol. 2014;8(1):26–31. doi: 10.1590/S1980-57642014DN81000005 29213876 PMC5619445

[21] Lezak MD, Howieson DB, Bigler ED, Tranel D. Neuropsychological Assessment. 5 ed. Oxford University Press; 2012.

[22] Corsi PM. Human memory and the medial temporal region of the brain. Montreal: McGill University; 1973;34.

[23] Morris JC, Heyman A, Mohs RC, Hughes JP, van Belle G, Fillenbaum G, et al. The Consortium to Establish a Registry for Alzheimer’s Disease (CERAD). part i. clinical and neuropsychological assessment of Alzheimer’s disease. Neurology. 1989;39(9):1159–65. doi: 10.1212/wnl.39.9.1159 2771064

[24] Sunderland T, Hill JL, Mellow AM, Lawlor BA, Gundersheimer J, Newhouse PA, et al. Clock drawing in Alzheimer’s disease. a novel measure of dementia severity. J Am Geriatr Soc. 1989;37(8):725–9. doi: 10.1111/j.1532-5415.1989.tb02233.x 2754157

[25] Marini A, Andreetta S. A combined psycholinguistic and neurocognitive perspective: age-related effects on language production. Cognition, Language and Aging. USA: East Carolina University; 2016:55–79.

[26] Arbuckle TY, Gold DP. Aging, inhibition, and verbosity. J Gerontol. 1993;48(5):225–32. 10.1093/geronj/48.5.p225 8366267

[27] Thompson CK, Mack JE. Grammatical Impairments in PPA. Aphasiology. 2014;28(8–9):1018–37. doi: 10.1080/02687038.2014.912744 25642014 PMC4306464

[28] Carthery-Goulart MT, de Oliveira R, de Almeida IJ, Campanha A, da Silva Souza D, Zana Y. Sentence comprehension in primary progressive aphasia: a study of the application of the Brazilian version of the test for the reception of grammar (TROG2-Br). Front Neurol. 2022;13:815227. 35651345 10.3389/fneur.2022.815227 PMC9149594

[29] Henry ML, Gorno-Tempini ML. The logopenic variant of primary progressive aphasia. Curr Opin Neurol. 2010;23(6):633–7. 20852419 10.1097/WCO.0b013e32833fb93e PMC3201824

[30] Charles D, Olm C, Powers J, Ash S, Irwin DJ, McMillan CT, et al. Grammatical comprehension deficits in non-fluent/agrammatic primary progressive aphasia. J Neurol Neurosurg Psychiatry. 2014;85(3):249–56. doi: 10.1136/jnnp-2013-305749 24039027 PMC3925677

[31] Roelofs A. A neurocognitive computational account of word production, comprehension, and repetition in primary progressive aphasia. Brain Lang. 2022;227:105094. doi: 10.1016/j.bandl.2022.105094 35202892

[32] Leyton CE, Savage S, Irish M, Schubert S, Piguet O, Ballard KJ, et al. Verbal repetition in primary progressive aphasia and Alzheimer’s disease. J Alzheimers Dis. 2014;41(2):575–85. doi: 10.3233/JAD-132468 24662100

[33] Ogar JM, Dronkers NF, Brambati SM, Miller BL, Gorno-Tempini ML. Progressive nonfluent aphasia and its characteristic motor speech deficits. Alzheimer Dis Assoc Disord. 2007;21(4):S23–30. doi: 10.1097/WAD.0b013e31815d19fe 18090419

[34] Lukic S, Mandelli ML, Welch A, Jordan K, Shwe W, Neuhaus J, et al. Neurocognitive basis of repetition deficits in primary progressive aphasia. Brain Lang. 2019;194:35–45. doi: 10.1016/j.bandl.2019.04.003 31055171 PMC6669076

[35] Henry ML, Grasso SM. Assessment of individuals with primary progressive aphasia. Semin Speech Lang. 2018;39(3):231–41. doi: 10.1055/s-0038-1660782 29933490 PMC6464628

[36] Tee BL, Gorno-Tempini ML. Primary progressive aphasia: a model for neurodegenerative disease. Curr Opin Neurol. 2019;32(2):255–65. doi: 10.1097/WCO.0000000000000673 30694922 PMC6602793

[37] Peristeri E, Messinis L, Kosmidis M, Nasios G, Mentis A, Siokas V. The impact of primary progressive aphasia on picture naming and general language ability. Cogn Behav Neurol. 2021;34(3):188–99. 34473670 10.1097/WNN.0000000000000275

[38] Migliaccio R, Boutet C, Valabregue R, Ferrieux S, Nogues M, Lehéricy S, et al. The brain network of naming: a lesson from primary progressive aphasia. PLoS One. 2016;11(2):e0148707. doi: 10.1371/journal.pone.0148707 26901052 PMC4764674

[39] Hillis AE, Oh S, Ken L. Deterioration of naming nouns versus verbs in primary progressive aphasia. Ann Neurol. 2004;55(2):268–75. doi: 10.1002/ana.10812 14755731

[40] Thompson CK, Lukic S, King MC, Mesulam MM, Weintraub S. Verb and noun deficits in stroke-induced and primary progressive aphasia: the Northwestern Naming Battery(). Aphasiology. 2012;26(5):632–55. doi: 10.1080/02687038.2012.676852 23188949 PMC3505449

[41] de Lira JO, Minett TSC, Bertolucci PHF, Ortiz KZ. Evaluation of macrolinguistic aspects of the oral discourse in patients with Alzheimer’s disease. Int Psychogeriatr. 2019;31(9):1343–53. doi: 10.1017/S1041610218001758 30520395

[42] Wilson SM, Henry ML, Besbris M, Ogar JM, Dronkers NF, Jarrold W, et al. Connected speech production in three variants of primary progressive aphasia. Brain. 2010;133(Pt 7):2069–88. doi: 10.1093/brain/awq129 20542982 PMC2892940

[43] Haley KL, Jacks A, Jarrett J, Ray T, Cunningham KT, Gorno-Tempini ML, et al. Speech metrics and samples that differentiate between nonfluent/agrammatic and logopenic variants of primary progressive aphasia. J Speech Lang Hear Res. 2021;64(3):754–75. doi: 10.1044/2020_JSLHR-20-00445 33630653 PMC8608203

[44] Rofes A, de Aguiar V, Ficek B, Wendt H, Webster K, Tsapkini K. The role of word properties in performance on fluency tasks in people with primary progressive aphasia. J Alzheimers Dis. 2019;68(4):1521–34. doi: 10.3233/JAD-180990 30909222 PMC6548439

[45] Libon DJ, McMillan C, Gunawardena D, Powers C, Massimo L, Khan A, et al. Neurocognitive contributions to verbal fluency deficits in frontotemporal lobar degeneration. Neurology. 2009;73(7):535–42. doi: 10.1212/WNL.0b013e3181b2a4f5 19687454 PMC2730797

[46] Laisney M, Matuszewski V, Mézenge F, Belliard S, de la Sayette V, Eustache F, et al. The underlying mechanisms of verbal fluency deficit in frontotemporal dementia and semantic dementia. J Neurol. 2009;256(7):1083–94. doi: 10.1007/s00415-009-5073-y 19363702

[47] Riello M, Frangakis CE, Ficek B, Webster KT, Desmond JE, Faria AV, et al. Neural correlates of letter and semantic fluency in primary progressive aphasia. Brain Sci. 2021;12(1):1. doi: 10.3390/brainsci12010001 35053745 PMC8773895

[48] van den Berg E, Dijkzeul JCM, Poos JM, Eikelboom WS, van Hemmen J, Franzen S, et al. Differential linguistic features of verbal fluency in behavioral variant frontotemporal dementia and primary progressive aphasia. Appl Neuropsychol Adult. 2024;31(4):669–77. doi: 10.1080/23279095.2022.2060748 35416098 PMC10069460

[49] Barbieri E, Litcofsky KA, Walenski M, Chiappetta B, Mesulam M-M, Thompson CK. Online sentence processing impairments in agrammatic and logopenic primary progressive aphasia: evidence from ERP. Neuropsychologia. 2021;151:107728. doi: 10.1016/j.neuropsychologia.2020.107728 33326758 PMC7875464

[50] Faria AV, Crinion J, Tsapkini K, Newhart M, Davis C, Cooley S, et al. Patterns of dysgraphia in primary progressive aphasia compared to post-stroke aphasia. Behav Neurol. 2013;26(1–2):21–34. doi: 10.3233/BEN-2012-110237 22713396 PMC3620674

[51] Lo Monaco MR, Di Tella S, Anzuino I, Ciccarelli N, Silveri MC. Writing errors in primary progressive aphasia. Appl Neuropsychol Adult. 2022;29(4):802–9. 32905710 10.1080/23279095.2020.1811707

[52] Rapp B, Glucroft B. The benefits and protective effects of behavioural treatment for dysgraphia in a case of primary progressive aphasia. Aphasiology. 2009;23(2):236–65. doi: 10.1080/02687030801943054 21603153 PMC3096931

[53] Teichmann M, Sanches C, Moreau J, Ferrieux S, Nogues M, Dubois B, et al. Does surface dyslexia/dysgraphia relate to semantic deficits in the semantic variant of primary progressive aphasia?. Neuropsychologia. 2019;135:107241. doi: 10.1016/j.neuropsychologia.2019.107241 31682928

[54] Rapp B, Purcell J, Hillis AE, Capasso R, Miceli G. Neural bases of orthographic long-term memory and working memory in dysgraphia. Brain. 2016;139(Pt 2):588–604. doi: 10.1093/brain/awv348 26685156 PMC4805091

[55] Graham NL. Dysgraphia in primary progressive aphasia: characterisation of impairments and therapy options. Aphasiology. 2014;28(8–9):1092–111. doi: 10.1080/02687038.2013.869308

[56] Zanichelli L, Fonseca RP, Ortiz KZ. Influence of age and schooling in written discourse of healthy adults. Psicologia: Reflexão e Crítica. 2020;33(1):10. 10.1186/s41155-020-00148-7 PMC7280393 32514630

[57] Botha H, Duffy JR, Strand EA, Machulda MM, Whitwell JL, Josephs KA. Nonverbal oral apraxia in primary progressive aphasia and apraxia of speech. Neurology. 2014;82(19):1729–35. doi: 10.1212/WNL.0000000000000412 24727315 PMC4032207

[58] Botha H, Josephs KA. Primary progressive aphasias and apraxia of speech. Continuum (Minneap Minn). 2019;25(1):101–27. doi: 10.1212/CON.0000000000000699 30707189 PMC6548538

[59] De Luccia G, Ortiz KZ. Ability of aphasic individuals to perform numerical processing and calculation tasks. Arq Neuropsiquiatr. 2014;72(3):197–202. doi: 10.1590/0004-282X20130250 24676436

[60] Ramanan S, Irish M, Patterson K, Rowe JB, Gorno-Tempini ML, Lambon Ralph MA. Understanding the multidimensional cognitive deficits of logopenic variant primary progressive aphasia. Brain. 2022;145(9):2955–66. doi: 10.1093/brain/awac208 35857482 PMC9473356

